# Herb-Drug Interaction: A Case Study of Effect of Ginger on the Pharmacokinetic of Metronidazole in Rabbit

**DOI:** 10.4103/0250-474X.41462

**Published:** 2008

**Authors:** J. M. Okonta, M. Uboh, W. O. Obonga

**Affiliations:** Department of Clinical Pharmacy and Pharmacy Management, University of Nigeria, Nsukka, Nigeria; 1 Department of Pharmaceutical Chemistry, University of Nigeria, Nsukka, Nigeria

**Keywords:** Metronidazole, ginger, herb-drug interaction, pharmacokinetics

## Abstract

The effect of ginger on the pharmacokinectic of metronidazole was studied using rabbits in a crossover study method. The relevance of this study borders on the wide use of ginger for culinary and phytotherapeutic purposes, and metronidazole that is commonly used for every gastrointestinal complain in our communities without prescription. Ginger significantly increased the absorption and plasma half-life, and significantly decreased the elimination rate constant and clearance of metronidazole (*P*<0.05). Thus, in clinical practice, the patients should be advised on the serious implication of using both items together.

*Zingiber officinalis,* Roscoe, is a rhizome that is widely used as culinary herb and herbal remedy for some common ailments. It is used as carminative, antipyretic, antiemetic in pregnancy and as anticancer adjunct^1^. It also ameliorates motion sickness and it is a known thromboxane synthesis and platelet aggregation inhibitors, and diaphoretic agent^1^. It contains about 1-2% of volatile oil and 5-8% of resinous matter, starch and mucilage^1^. The volatile oil contains monoterpenes, sesquiterpenes and sesquiterpene alcohol zingiberol^1^, gingerol and shagoals. Most of the pharmacologically active constituents reside in the volatile oils. Gingerols have analgesic, sedative, antipyretic and antibacterial effects both *in vitro* and *in vivo*^2^–^4^. Shagoal has antiemetic, antispasmodic, anxiolytic and anticonvulsant activity^5^–^6^.

Metronidazole is a potent protocidal and trichomonacidal agent and has good activity against intestinal amebiasis^7^, and many gastrointestinal complaints^8^–^9^. Its effective plasma concentration is 10 μg/ml^10^, which occurs within 1 to 2 h post administration orally after a single 400 mg dose^11^.

The use of complimentary or alternative remedies is on the increase globally because most people believe that the natural agents are safer than the conventional therapeutic agents. Ginger is known to improve blood circulation and bioavailability of other herbs^5^–^12^ when concurrently administered or co-formulated. In Nigeria, the indiscriminate use of unprescribed metronidazole for diverse gastrointestinal problems is high. Given that ginger is also widely used by a vast majority of the populace for various ailments including gastrointestinal, there is a high probability of concomitant consumption of ginger and metronidazole. Our present study therefore attempts at elucidating the possible herb-drug interaction between metronidazole and ginger viz-a-viz the effect of ginger on the pharmacokinetics of metronidazole.

Metronidazole tablets (200 mg) were purchased from a registered pharmacy in Nsukka, metronidazole pure powder was a gift from Evans, Nigeria. Sulphuric acid (Sigma, Germany) and methanol (Fluka, Germany) were analytical grade. Five healthy locally bred white rabbits (1.0-1.8 kg) of 3 females and 2 males were used for the study. They were also separated into cages according to gender to avoid conception before and during the study. They were fasted for 12 h before the commencement of the study but had access to water *ad libitum*. The animals were handled according to internationally and locally approved protocol for handling animal based experiment.

Fresh rhizomes of ginger purchased from Nsukka market were washed, peeled and 10 g weighed out and mashed with mortar and pestle. This was then extracted with 0.1 l of pure water and boiled for 5 min at 100°. The extract was filtered and administered 1 ml/kg orally.

Drug-food interaction study design consisted of two-phase or crossover study method. In phase one, the five healthy local strain rabbits were each given 3 mg/kg per oral metronidazole and 0.5 ml blood sample from their marginal veins were withdrawn over 24 h (0, 1, 2, 4, 8 and 24 h). The withdrawn blood samples were analyzed immediately. The animals were allowed a washout period of 2 weeks. In the phase two, all the five rabbits were given 1 ml/kg of ginger extract per oral daily for 3 days and immediately given 3 mg/kg metronidazole per oral on the third day. Blood samples, 0.5 ml, were collected at the same time intervals of 0, 1, 2, 4, 8 and 24 h, and the samples analyzed immediately.

Extraction of metronidazole from the blood samples was carried out following the method described by Ofoefule *et al*^13^. Blood samples (0.5 ml) were mixed with 5 ml of 0.1 N H_2_SO_4_ in methanol and centrifuged for 15 min at 3000 g. The supernatants were separated and analyzed spectrophotometrically at 323 nm for metronidazole against plasma blank containing no drug.

For the generated data on metronidazole and ginger interaction to be analyzed, we assumed that the kinetics of metronidazole elimination was linear. The data was represented in a plasma level-time curve from where the area under time curve (AUC_0-24h_) was calculated using Trapezoid rule. The maximum concentration (C_max_) and maximum time (T_max_) were obtained directly from generated data. The elimination constant (K) and half-life (t_1/2_) were determined from the semi-log plot of the data. The clearance (CL) and apparent volume of distribution (V_d_) of the drug in the animals were calculated from the equations, CL= (0.7×V_d_)/t_1/2_…1, V_d_= D_b_°/C_p_°…2 and AUC_∞_ = AUC_0-24h_+C_24h_/k…3, where, D_b_° is the administered dose of drug; C_p_° is the initial plasma concentration of drug obtained at intercept of semi log plot of plasma drug sample and C_24hour_ is the plasma drug concentration at 24^th^ h.

The results were analyzed statistically using analysis of variance (ANOVA) and the student's t-test. Means that differed significantly were identified using the least square difference (LSD) post-hoc test at 95% confidence interval (p<0.05).

The mean plasma concentration-time curve for metronidazole (3 mg/kg) alone and metronidazole after once a day administration of oral ginger extract for 3 days (1 ml/kg) was shown in fig. 1. The study was done for 24 h since the half-life of metronidazole is 8 h^11^. The pharmacokinetic parameters were shown in Table 1. The T_max_ and C_max,_ AUC_0-24h_ and AUC∞ showed significant difference (p<0.05) between the metronidazole alone and metronidazole plus ginger treated groups. The peak (C_max_) in the plasma concentration-time curve of metronidazole plus ginger occurred at about 4 h while the metronidazole alone was 2 h post administration, indicating that ginger may have caused delay in the rate of absorption of oral metronidazole but enhanced the extent of absorption considering the significant difference between the AUC_0-24h_ of metronidazole alone and metronidazole plus ginger group. This is shown in the relatively prolonged T_1/2_ (h) from 8.8 h to 12.7 h while that of human has been reported to be about 8 h^11^. The metronidazole clearance was reduced from 1.648 to0.558 ml/kg.h (p<0.05) and the elimination rate was also reduced from 0.079 to 0.054 h^-1^ (p<0.05).

**Fig. 1:**
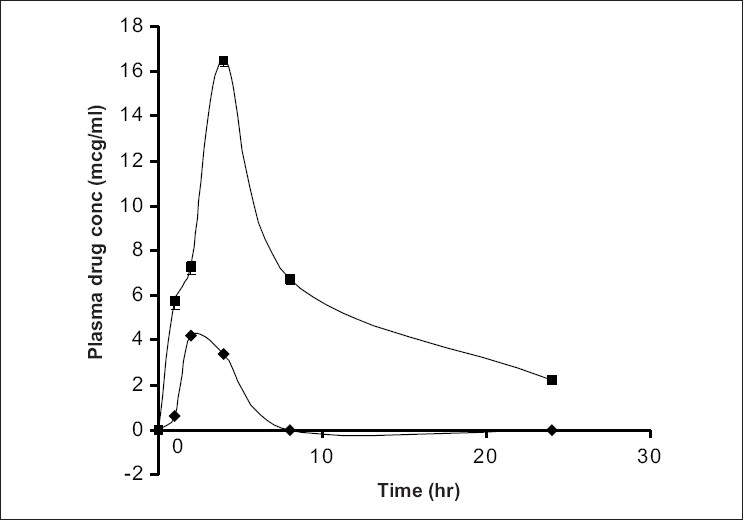
Effects of ginger on plasma metronidazole concentration. Effect of Ginger (G) at 1 ml/kg concentration on the plasma levels of metronodazole (MTN 3 mg/kg). MTZ alone (–♦–) and MTZ in the presence of Ginger (–■–).

**TABLE 1:** PHARMACOKINETIC PARAMETERS OF METRONIDAZOLE ON COADMINISTERED WITH GINGER

Parameter	MTZ alone	MTZ + Ginger
C_max_ (μg/ml)	4.23±0.45	16.50±1.03*
T_max_ (hour)	2.0±0.07	4.0±0.23*
AUC_0-24h_ (μg/h.ml)	17.16±2.01	151.1±3.17*
AUC_-_ (μg/h.ml)	1 7.16±1.09	192.0±12.04*
Kel (h^-1^)	0.079±0.003	0.054±0.001
CL (ml/kg.h)	1.648±0.42	0.558±0.11
T_1/2_ (h)	8.6±0.76	12.7±1.05*
Vd (ml/kg^1^)	20.48±2.04	10.23±2.13

* p<0.05 significant to the parameter of MTZ alone.

* N/B. MTZ means metronidazole

The observations from our data showed that ginger, as a spice, condiment and phytotherapeutic agent, has definite and significant effects on the absorption kinetics of metronidazole. Since the peak plasma concentration of metronidazole when co administered with ginger occurred at about two hours later than that of metronidazole alone, its an indication that ginger may have caused delay in the rate of absorption of oral metronidazole but enhanced the extent of absorption considering the significant difference between the AUC_0-24h_ of metronidazole alone and metronidazole plus ginger group. Ginger is known to improve blood circulation and bioavailability of some herbs^5^,^12^ when concurrently administered or co-formulated. It has also been reported to have spasmolytic effect and do cause smooth muscles relaxation^6^. These effects may have caused a reduction in gastric emptying, gastrointestinal motility and increased the blood circulation to the gastrointestinal tract thereby facilitating the increased absorption of metronidazole.

The elimination rate constant (k_el_) and clearance of a drug indicates the proportion of that drug that is removed from the body^14^ and half-life is a reciprocal function of these. As the co administration of ginger and metronidazole reduced the elimination rate constant and the clearance of the drug especially in linear kinetic, it invariably caused prolongation of the half-life. The liver is the main site of metabolism of metronidazole and about 50% of the drug is cleared from the systemic circulation by the liver^9^; since ginger decreased the clearance of the drug, it may be that ginger altered the metabolism of the drug by the liver.

These pharmacokinectic effects of ginger must be cautiously considered if the metronidazole must be used by a patient that consumes ginger in whatever form as the peak plasma concentrations of approximately 5 to 10 μg/ml are achieved within 1-3 hours after single doses of 250 and 500 mg of oral metronidazole^13^, and the co administration of ginger and metronidazole at 3 mg/kg increased the peak plasma concentration from 4.23 to 16.50 μg/ml, which is about four folds.

In conclusion, this study revealed that ginger could cause increase in the bioavailability and half-life, and decrease in the clearance and elimination rate constant of metronidazole per oral. This may pose a negative implication in clinical practice as toxicity of metronidazole may easily be reached especially during multiple dosing because of the possibility of drug accumulation.
